# Supplementary material for: The impact of future time perspective on academic achievement: Mediating roles of academic burnout and engagement
Source: PLoS One. 2025 Jan 31;20(1):e0316841. doi: 10.1371/journal.pone.0316841 (PMC11785328; doi:10.1371/journal.pone.0316841)
Supplement: S1 Appendix — (DOCX) [file pone.0316841.s001.docx]

**S1 Appendix. Zimbardo Future Time Perspective Scale**

*Note*: The original items of the questionnaire were administered in Chinese, the participants’ native language. For publication purposes, the items have been translated into English.

**Instruction:** *Please indicate your agreement with each statement by selecting a score from 1 (Strongly Disagree) to 5 (Strongly Agree).*

**Table S1.** Zimbardo Future Time Perspective Scale (5 items)

| **No.** | **Item** | **Score (1-5)** |
| --- | --- | --- |
| 1 | I complete projects on time by making steady progress. | 1□ 2□ 3□ 4□ 5□ |
| 2 | Meeting tomorrow’s deadlines and doing other necessary work comes before tonight’s play. | 1□ 2□ 3□ 4□ 5□ |
| 3 | When I want to achieve something, I set goals and consider specific means for reaching those goals. | 1□ 2□ 3□ 4□ 5□ |
| 4 | I keep working at difficult, uninteresting tasks if they will help me get ahead. | 1□ 2□ 3□ 4□ 5□ |
| 5 | I meet my obligations to friends and authorities on time. | 1□ 2□ 3□ 4□ 5□ |
