# Supplementary material for: The impact of future time perspective on academic achievement: Mediating roles of academic burnout and engagement
Source: PLoS One. 2025 Jan 31;20(1):e0316841. doi: 10.1371/journal.pone.0316841 (PMC11785328; doi:10.1371/journal.pone.0316841)
Supplement: S2 Appendix — (DOCX) [file pone.0316841.s002.docx]

**S2 Appendix. Academic Burnout Scale**

*Note*: The original items of the questionnaire were administered in Chinese, the participants’ native language. For publication purposes, the items have been translated into English.

**Instruction:** *Please rate the extent to which you experience each item on a scale from 1 (not at all) to 5 (very much).*

*Note: Items marked with an asterisk (*) are reverse-scored.*

**Table S2.** Learning Burnout of Undergraduate Scale (20 items)

| **Emotional Exhaustion** | | |
| --- | --- | --- |
| **No.** | **Item** | **Score (1-5)** |
| 1 | I feel like the things I’m learning are pointless. | 1□ 2□ 3□ 4□ 5□ |
| 2 | When I wake up in the morning, I feel tired just thinking about facing a day of studying. | 1□ 2□ 3□ 4□ 5□ |
| 3 | It is hard for me to stay interested in studying for the long term. | 1□ 2□ 3□ 4□ 5□ |
| 4 | After a long day of studying, I feel completely worn out. | 1□ 2□ 3□ 4□ 5□ |
| 5 | I feel bored with studying. | 1□ 2□ 3□ 4□ 5□ |
| 6 | I often feel sleepy while I’m studying. | 1□ 2□ 3□ 4□ 5□ |
| 7 | I want to get involved in learning, but I always end up feeling bored. | 1□ 2□ 3□ 4□ 5□ |
| 8 | I’m constantly exhausted from all the exams. | 1□ 2□ 3□ 4□ 5□ |
| **Misbehavior** | | |
| **No.** | **Item** | **Score (1-5)** |
| *1 | I have my own learning methods and plans, and I can actually stick to them | 1□ 2□ 3□ 4□ 5□ |
| *2 | So far, my studies in universities have really shown my abilities. | 1□ 2□ 3□ 4□ 5□ |
| 3 | I rarely study outside of class. | 1□ 2□ 3□ 4□ 5□ |
| 4 | I don’t have much patience when it comes to studying. | 1□ 2□ 3□ 4□ 5□ |
| 5 | I only crack open a book when exams are coming up. | 1□ 2□ 3□ 4□ 5□ |
| 6 | I hardly ever plan or schedule my study time. | 1□ 2□ 3□ 4□ 5□ |
| **Inefficacy** | | |
| **No.** | **Item** | **Score (1-5)** |
| *1 | Mastering my course material comes pretty easily to me. | 1□ 2□ 3□ 4□ 5□ |
| *2 | When I’m studying, I can handle my emotions calmly. | 1□ 2□ 3□ 4□ 5□ |
| *3 | I feel confident and capable in my university courses. | 1□ 2□ 3□ 4□ 5□ |
| *4 | I find my major interesting. | 1□ 2□ 3□ 4□ 5□ |
| *5 | Earning my bachelor’s degree doesn’t seem too challenging to me. | 1□ 2□ 3□ 4□ 5□ |
| *6 | I feel full of energy when I’m studying. | 1□ 2□ 3□ 4□ 5□ |
