# Supplementary material for: The impact of future time perspective on academic achievement: Mediating roles of academic burnout and engagement
Source: PLoS One. 2025 Jan 31;20(1):e0316841. doi: 10.1371/journal.pone.0316841 (PMC11785328; doi:10.1371/journal.pone.0316841)
Supplement: S3 Appendix — (DOCX) [file pone.0316841.s003.docx]

**S3 Appendix. Academic Engagement Scale**

*Note*: The original items of the questionnaire were administered in Chinese, the participants’ native language. For publication purposes, the items have been translated into English.

**Instruction:** *Please rate how often each of the following statements describes your experience. Use the scale below and mark each statement on a scale from 1 (never) to 7 (always).*

**Table S3.** Utrecht Work Engagement Scale for Students (17 items)

| **Vitality** | | |
| --- | --- | --- |
| **No.** | **Item** | **Score (1-7)** |
| 1 | I feel full of energy when studying or attending classes. | 1□ 2□ 3□ 4□ 5□ 6□ 7□ |
| 2 | When I wake up in the morning, I feel motivated to study. | 1□ 2□ 3□ 4□ 5□ 6□ 7□ |
| 3 | I’m satisfied with my studies. | 1□ 2□ 3□ 4□ 5□ 6□ 7□ |
| 4 | I can study for extended periods with full energy. | 1□ 2□ 3□ 4□ 5□ 6□ 7□ |
| 5 | Even if I feel mentally exhausted, I can recover quickly and keep studying. | 1□ 2□ 3□ 4□ 5□ 6□ 7□ |
| 6 | I’m able to keep going with energy, even if my studies aren’t going well. | 1□ 2□ 3□ 4□ 5□ 6□ 7□ |
| **Dedication** | | **Score (1-7)** |
| 1 | I feel energized when I’m studying. | 1□ 2□ 3□ 4□ 5□ 6□ 7□ |
| 2 | I believe learning is valuable and meaningful. | 1□ 2□ 3□ 4□ 5□ 6□ 7□ |
| 3 | Time flies when I’m studying. | 1□ 2□ 3□ 4□ 5□ 6□ 7□ |
| 4 | I find studying interesting. | 1□ 2□ 3□ 4□ 5□ 6□ 7□ |
| 5 | Learning sparks my intellectual curiosity. | 1□ 2□ 3□ 4□ 5□ 6□ 7□ |
| 6 | I feel happy when I’m fully focused on my studies. | 1□ 2□ 3□ 4□ 5□ 6□ 7□ |
| **Absorption** | | **Score (1-7)** |
| 1 | When I’m studying, I get so focused that I lose track of what’s happening around me. | 1□ 2□ 3□ 4□ 5□ 6□ 7□ |
| 2 | I can concentrate easily when I’m studying. | 1□ 2□ 3□ 4□ 5□ 6□ 7□ |
| 3 | I enjoy exploring new questions that come up during my studies. | 1□ 2□ 3□ 4□ 5□ 6□ 7□ |
| 4 | When I study, I sometimes reach a state where I lose awareness of myself. | 1□ 2□ 3□ 4□ 5□ 6□ 7□ |
| 5 | I can study without being easily distracted. | 1□ 2□ 3□ 4□ 5□ 6□ 7□ |
