# Supplementary material for: The impact of future time perspective on academic achievement: Mediating roles of academic burnout and engagement
Source: PLoS One. 2025 Jan 31;20(1):e0316841. doi: 10.1371/journal.pone.0316841 (PMC11785328; doi:10.1371/journal.pone.0316841)
Supplement: S4 Appendix — (DOCX) [file pone.0316841.s004.docx]

**S4 Appendix. Summary of Factor Loading, Goodness-of-Fit Indices, and Reliability for Measurement Scales**

**Table S4.** Factor Loadings, Model Fit, and Reliability of Study Scales

| **Scale** | **Factor** | **Standardized Factor Loadings Range** | **Goodness of Fit**  **(CFI, TLI, RMSEA, SRMR)** | **Reliability**  **(α)** |
| --- | --- | --- | --- | --- |
| Future Time Perspective | Future Time Perspective | 0.61 – 0.73 | CFI = 0.98, TLI = 0.97,  RMSEA = 0.06, SRMR = 0.02 | α = 0.75 |
| Academic Burnout | Emotional Exhaustion | 0.62 – 0.84 | CFI = 0.90, TLI = 0.89,  RMSEA = 0.07, SRMR = 0.04 | α = 0.86 |
|  | Misbehavior | 0.74 – 0.78 |  | α = 0.78 |
|  | Inefficacy | 0.59 – 0.73 |  | α = 0.73 |
| Academic Engagement | Academic engagement | 0.62 – 0.88 | CFI = 0.94, TLI = 0.93,  RMSEA = 0.08, SRMR = 0.03 | α = 0.89 |
